# Overexpression of satellite RNAs in heterochromatin induces chromosomal instability and reflects drug sensitivity in mouse cancer cells

**DOI:** 10.1038/s41598-022-15071-3

**Published:** 2022-06-29

**Authors:** Sawako Tamaki, Koichi Suzuki, Iku Abe, Yuhei Endo, Nao Kakizawa, Fumiaki Watanabe, Masaaki Saito, Shingo Tsujinaka, Yasuyuki Miyakura, Satoshi Ohta, Kenji Tago, Ken Yanagisawa, Fumio Konishi, Toshiki Rikiyama

**Affiliations:** 1 Department of Surgery, Saitama Medical Center, Jichi Medical University, 1-847, Amanuma-cho, Omiya-ku, Saitama, 330-8503 Japan; 2 Division of Structural Biochemistry, Department of Biochemistry, Jichi Medical University, 3311-1 Yakushiji, Shimotsuke-shi, Tochigi, 329-0498 Japan; 3 Nerima Hikarigaoka Hospital, 2-11-1, Hikarigaoka, Nerima-ku, Tokyo, 179-0072 Japan

**Keywords:** Cancer, Gastrointestinal cancer, Colorectal cancer

## Abstract

Overexpression of satellite RNAs in heterochromatin induces chromosomal instability (CIN) through the DNA damage response and cell cycle checkpoint activation. Although satellite RNAs may be therapeutic targets, the associated mechanisms underlying drug sensitivity are unknown. Here, we determined whether satellite RNAs reflect drug sensitivity to the topoisomerase I inhibitor camptothecin (CPT) via CIN induction. We constructed retroviral vectors expressing major satellite and control viruses, infected microsatellite stable mouse colon cancer cells (CT26) and MC38 cells harboring microsatellite instability, and assessed drug sensitivity after 48 h. Cells overexpressing satellite RNAs showed clear features of abnormal segregation, including micronuclei and anaphase bridging, and elevated levels of the DNA damage marker γH2AX relative to controls. Additionally, overexpression of satellite RNAs enhanced MC38 cell susceptibility to CPT [half-maximal inhibitory concentration: 0.814 μM (control) vs. 0.332 μM (MC38 cells with a major satellite), *p* = 0.003] but not that of CT26. These findings imply that MC38 cells, which are unlikely to harbor CIN, are more susceptible to CIN-induced CPT sensitivity than CT26 cells, which are characterized by CIN. Furthermore, CPT administration upregulated p53 levels but not those of p21, indicating that overexpression of major satellite transcripts likely induces CPT-responsive cell death rather than cellular senescence.

## Introduction

Many cancers exhibit “aneuploidy,” which is defined as the presence of an abnormal number of chromosomes in a cell^1^. Chromosomal instability (CIN) is characterized by a high frequency of chromosomal abnormalities, such as a gain or loss of entire chromosomes or large regions (aneuploidy), structural rearrangements, and localized abnormalities, such as amplifications and deletions^2,3^. It is estimated that 70–95% of cancers exhibit chromosomal abnormalities suggestive of CIN^4,5^. These alterations disrupt normal genome structure and function, increase the frequency of mutations, and cause epigenetic changes. Moreover, CIN increases diversity and heterogeneity, which in turn increases cancer malignancy and promotes drug resistance^6^.

The central part of the chromosome (the centromere) plays an important role in maintaining chromosomal stability, and its impairment facilitates abnormal segregation of chromosomes. The centromere comprises a 171-bp repetitive sequence called satellite DNA. Satellite α transcripts (SATs) are a heterogeneous population of noncoding RNAs transcribed from satellite DNA and include large swaths of repetitive sequences at the centromere and telomeres of a variety of eukaryotic chromosomes^7–9^. A landmark study in fission yeast demonstrated that the transcription of satellite RNAs is critical for the establishment and maintenance of pericentromeric heterochromatin^10^. Heterochromatic repetitive satellite RNAs are extensively transcribed in various human cancers^11^. Additionally, aberrant expression of satellite RNAs in cultured cells induces a DNA damage response, activates cell cycle checkpoints, and causes defects in chromosome segregation^12,13^. Furthermore, we have previously demonstrated that overexpression of SATs induced by retroviral expression vectors leads to changes in copy number at specific chromosomes^14^.

CIN is strongly associated with drug resistance, and numerous reports have indicated that CIN correlates with resistance to antineoplastic agents, such as taxol, in both tumor-derived cell lines and clinical settings^6,15–17^. However, excessive levels of CIN reportedly increase sensitivity to cytotoxic therapies, such as cisplatin and 5-fluorouracil (5-FU), in ovarian, rectal, and breast cancers^17–20^. A previous study showed that inducing whole-chromosome missegregation sensitizes transplanted human glioblastoma tumors to radiation treatment^21^. Furthermore, misexpression of genes at the centromere and kinetochore regions is reportedly associated with outcomes in cancer patients and their response to radiotherapy and chemotherapy^22^. Drug-induced genotoxicity leads to CIN, which may reduce tolerance to genotoxic stress in cancer cells. For example, replication forks are often stalled owing to replication stress, which causes genomic instability. Additionally, overexpression of satellite RNAs decreases the expression levels of proteins that play a role in stabilizing and repairing stalled replication forks^13^. Moreover, the formation of RNA–DNA hybrids at the replication fork prevents the re-stalling of replication forks. In cancer treatment, these types of genetic stress represent ideal targets for mediating cancer cell death.

DNA topoisomerase I inhibitors prevent the repair of single-stranded DNA breaks, leading to cancer cell death. Topoisomerase I inhibitors, such as irinotecan and topotecan, are camptothecin (CPT) analogs that damage DNA^23^. Topoisomerase I normally forms a DNA–topoisomerase I complex during DNA replication and translation, which relaxes the DNA double helix structure by creating a single-stranded DNA state and recombining the DNA. Topoisomerase inhibitors inhibit DNA recombination by stabilizing the DNA–topoisomerase I complex, ultimately leading to double-strand breaks and cell death. Zhang et al.^22^ reported that cancer cell lines with high gene expression at the centromere and kinetochore regions are more sensitive to topoisomerase I inhibitors than those with a low expression, and that genotoxicity decreases the survival of cells with CIN by reducing their tolerance to genotoxic stress. In this study, we determined whether satellite RNA-induced CIN enhances the sensitivity to topoisomerase I inhibitors, which prevent the repair of DNA single-strand breaks.

## Results

### Overexpression of satellite RNA and its effects on cell growth of cancer cell lines

**Figure 1:**
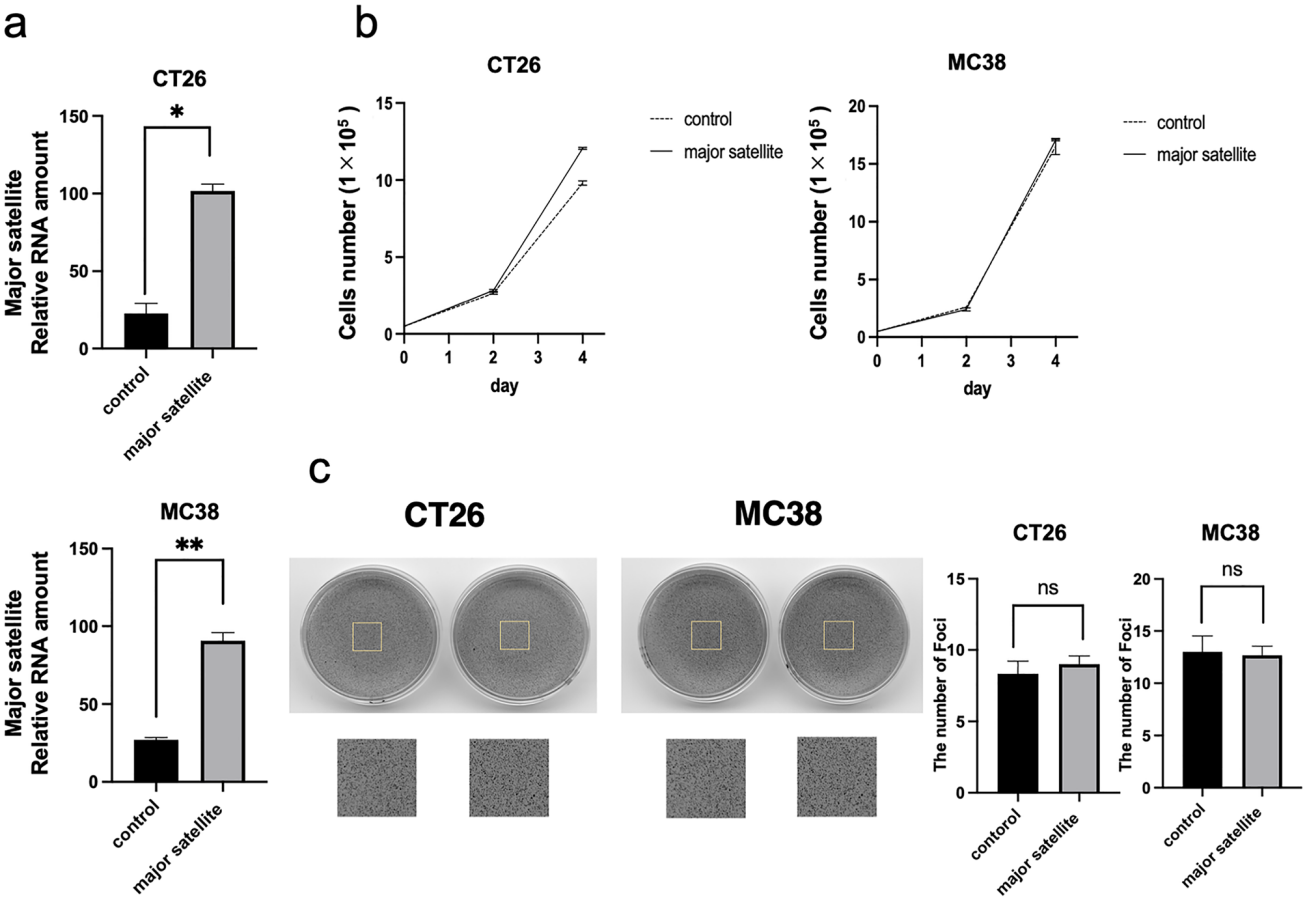
mSAT overexpression and cell proliferation. (**a**) Comparison of mSAT expression between cells infected by major satellite-expressing vectors and controls. Error bars indicate standard error, and the results of paired sample *t*-test are shown. (**b**) Cell proliferation curve. The cells were counted on days 2 and 4 after seeding, and the number of cells is shown. There was no difference in the proliferation of either cell line. (**c**) Soft agar assay. The anchorage-independent growth was confirmed, and the number of foci is shown. There was no difference between the mSAT-overexpressing cells and controls. *p < 0.05; **p < 0.01; ns, not significant.

To elucidate the effects of the overexpression of major satellite RNAs (mSATs) in cancer cell lines, we transduced mSATs using retroviral vectors into CT26 and MC38 cell lines. CT26 is a microsatellite stable (MSS) cancer cell line, whereas MC38 is a cell line with microsatellite instability (MSI). Successful transfection induced mSAT overexpression in these cells, with the expression levels of major satellites confirmed by qRT-PCR. The results verified the higher expression of mSATs in cells infected with major satellite-positive viruses relative to the control vector (control, 22.66 ± 11.24% vs. mSAT, 101.66 ± 4.41% in CT26 cells; 27.0 ± 1.15% vs. 90.66 ± 5.20% in MC38 cells; *p < 0.05, **p < 0.01, respectively) (Fig. 1a). We then investigated the effects of mSAT overexpression on cell growth and compared the number of mSAT-transfected and control CT 26 and MC38 cells after 4 days of culture. We observed gradual cell growth in both cell lines, although no difference in cell growth was observed between mSAT-transfected and control cells (Fig. 1b), as confirmed by soft agar colony formation assay (Fig. 1c).

### Overexpression of satellite RNA induces mitotic errors of chromosomes and DNA damage

**Figure 2:**
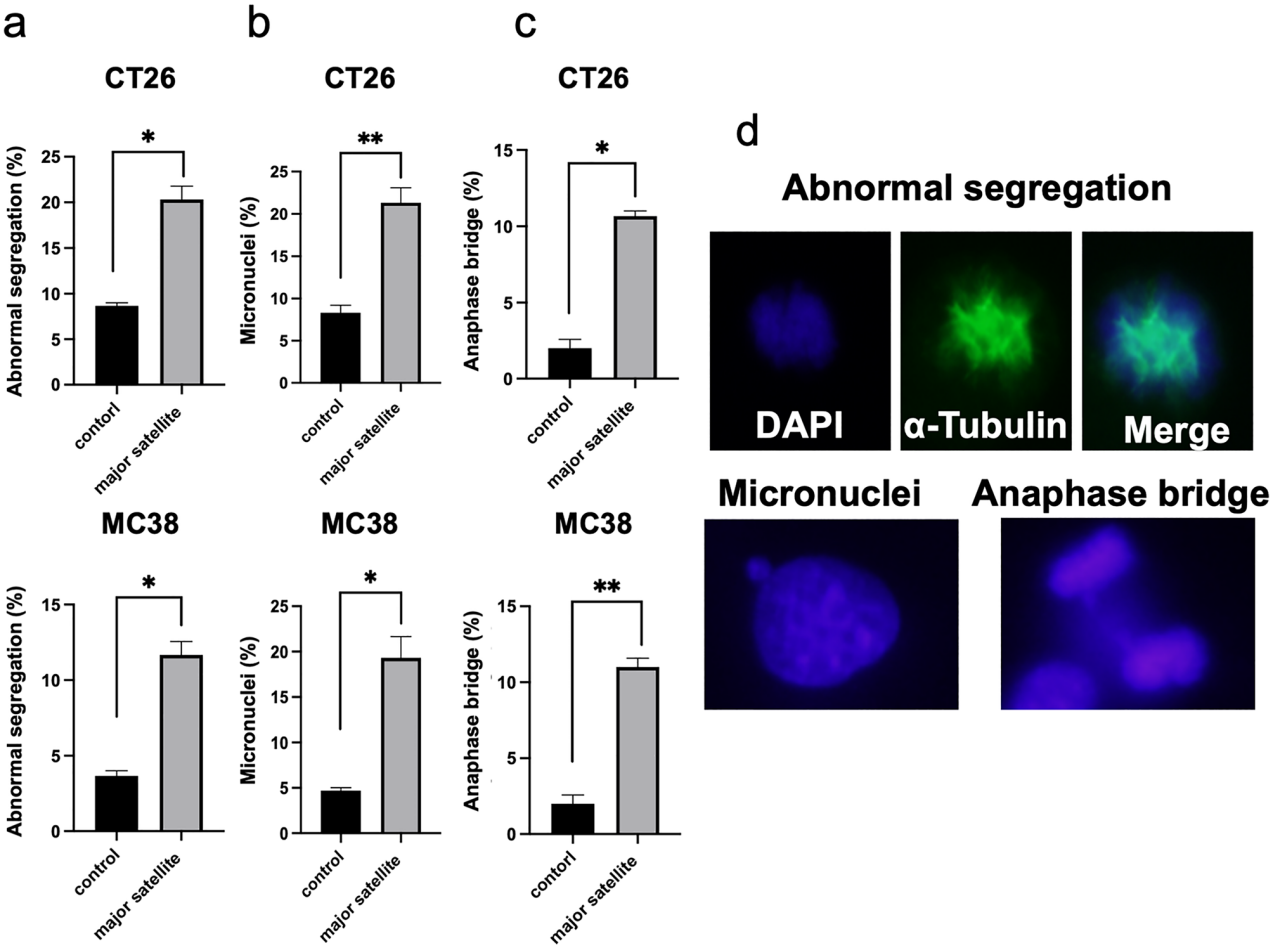
mSAT overexpression induces CIN. (**a–c**) Comparisons of the incidence of abnormal segregation, micronuclei, and anaphase bridging between mSAT-overexpressing cells and controls based on the evaluation of 100 mitotic cells. Error bars indicate standard error, and the results of paired sample *t*-test are shown. (**d**) Representative images of abnormal segregation, micronuclei, and anaphase bridging events in mSAT-overexpressing CT26 cells. *p < 0.05; **p < 0.01.

Next, we performed immunocytochemistry to detect mitotic errors that could lead to CIN. Evaluation of the proportion of cells with mitotic errors, including abnormal segregation of micronuclei and anaphase bridging, in > 100 mitotic cells/sample (representative of three independent experiments) revealed significant increases in abnormal segregations, micronuclei, and anaphase bridging events in both CT26 and MC38 cells infected with mSAT retroviruses compared with the controls (abnormal segregation: control, 8.67 ± 0.33% vs. mSAT, 20.33 ± 1.45% in CT26 cells; 4.00 ± 0.58% vs. 11.67 ± 0.88% in MC38 cells; *p < 0.05, Fig. 2a; micronuclei : control, 8.33 ± 0.88% vs. mSAT, 20.67 ± 1.76% in CT26 cells; 4.33 ± 0.33% vs. 19.33 ± 2.33% in MC38 cells; *p < 0.05, **p < 0.01, respectively, Fig. 2b; anaphase bridging: control, 2.00 ± 0.58% vs. mSAT 10.67 ± 0.33% in CT26 cells; 2.00 ± 0.58% vs. 11.0 ± 0.58% in MC38 cells; *p < 0.05, **p < 0.01, respectively, Fig. 2c). Figure 2d shows the representative images of abnormal segregation, including the micronuclei and anaphase bridging, in CT26 cells overexpressing mSATs.

**Figure 3:**
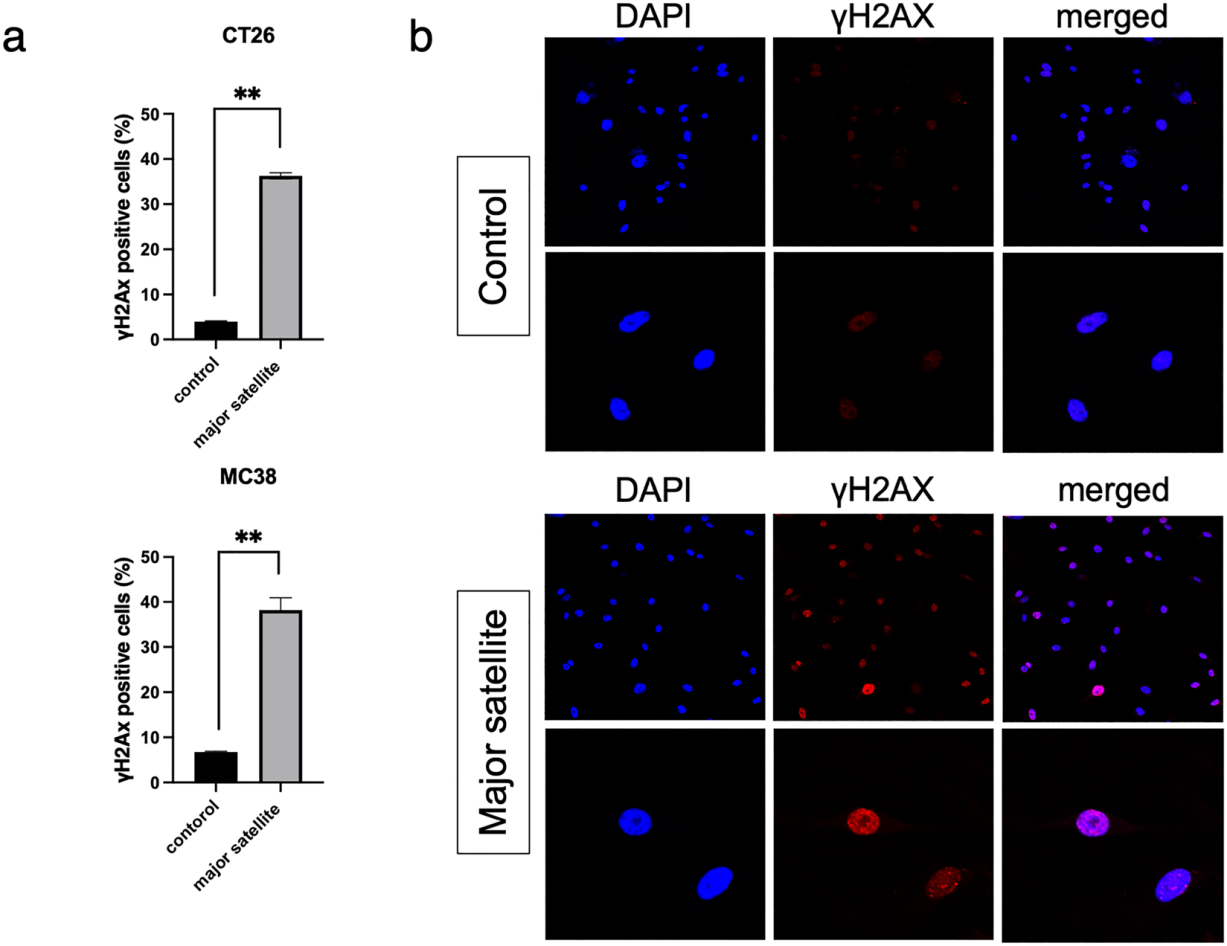
mSAT overexpression induces DNA damage. (**a**) DNA damage was evaluated according to the proportion of γH2AX-positive cells and increased in mSAT-overexpressing cells. Error bars indicate standard error, and the results of paired sample *t*-test are shown. (**b**) Image of γH2AX-positive cells in mSAT-overexpressing CT26 cells and controls. *p < 0.05, **p < 0.01.

We evaluated the DNA damage response based on the number of anti-γH2AX-positive cells and found that the proportion of anti-γH2AX-positive cells significantly increased in mSAT-overexpressing CT26 and MC38 cells compared with the control cells (control: 3.99 ± 0.13% vs. 36.26 ± 0.67% in CT26 cells; 6.78 ± 0.16% vs. 38.17 ± 2.77% in CT26 cells; *p < 0.05 and **p < 0.01, respectively, Fig. 3a). Additionally, Fig. 3b shows the images representing the evaluation of the incidence of anti-γH2AX-positive mSAT-overexpressing CT26 cells in three field of view per sample (representative of three independent experiments).

### Overexpression of satellite RNAs reflects drug sensitivity

**Figure 4:**
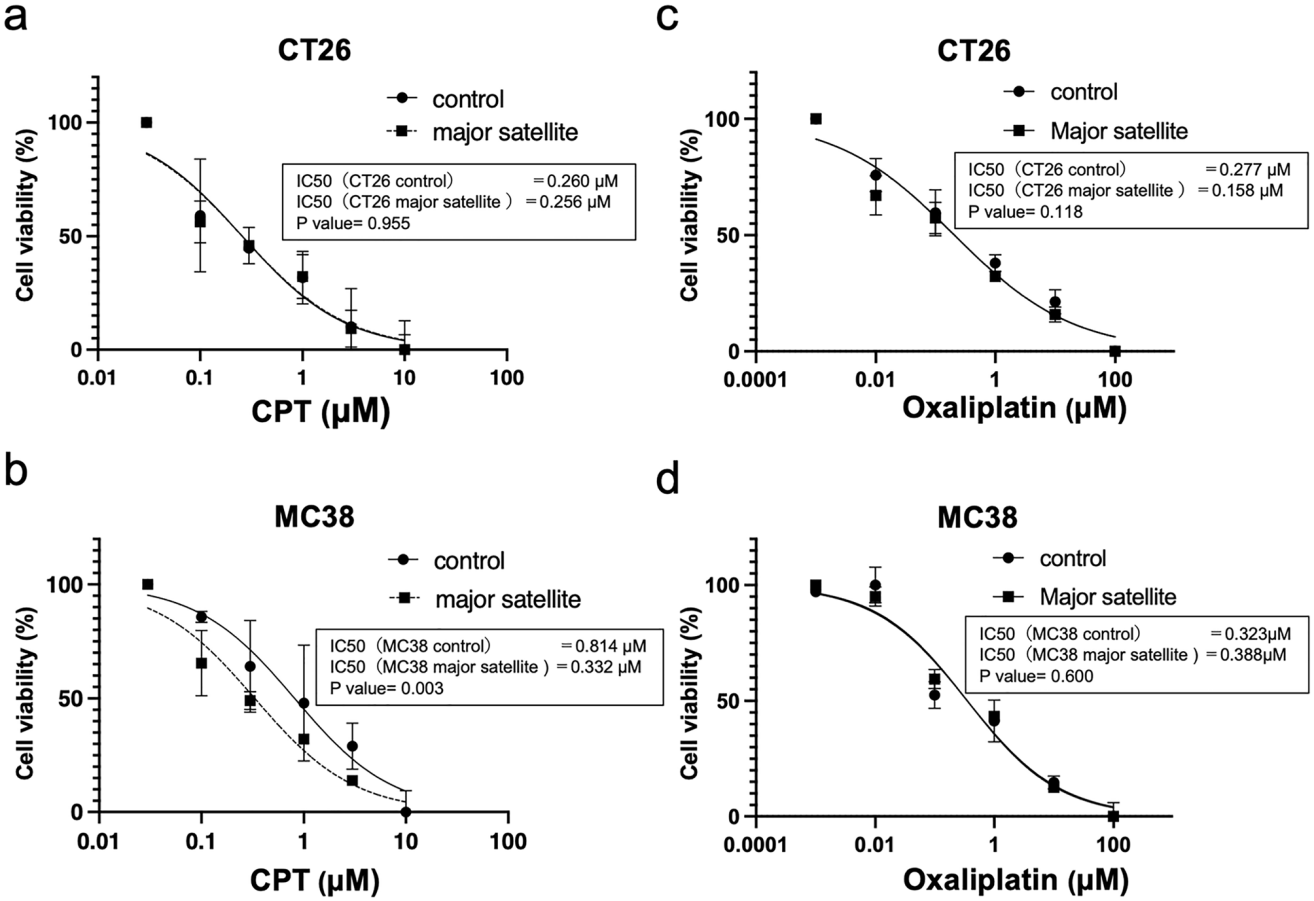
Overexpression of satellite RNAs reflects drug sensitivity. (**a**,**b**) Drug sensitivity was determined 48 h after CPT treatment. The graph shows the dose–response curves of the WST-1 assays at 48 h post-CPT treatment. IC_50_ values were obtained using a four-parameter logistic model, and dose–response curves were compared using a sum of squares *F* test. IC_50_ was significantly lower in MC 38 cells overexpressing mSAT than in those control. Overexpression of major satellites increased the sensitivity of MC38 cells to CPT. (**c**,**d**) Drug sensitivity was determined 24 h after oxaliplatin treatment. The graph shows the dose–response curves of the WST-1 assays at 24 h post-oxaliplatin treatment. IC_50_ values were not significantly different between the cells overexpressing mSAT and the control.

We determined drug sensitivity at 48 h after treatment with several doses of CPT and found that the growth of CT26 and MC 38 cells is inhibited in a dose-dependent manner. Notably, overexpression of mSAT increased the drug sensitivity of MC38 cells but not that of CT26 cells to CPT (Fig. 4a,b). In particular, MC38 sensitivity to CPT was significantly enhanced (IC_50_: control, 0.814 μM vs. mSAT, 0.332 μM in MC38 cells, *p* = 0.003), whereas that of CT26 cells had no significant difference (IC_50_: control, 0.260 μM vs. mSAT, 0.256 μM, *p* = 0.955). These findings imply that MC38 cells, which are unlikely to harbor CIN, are more susceptible to CIN-induced CPT sensitivity than CT26 cells, which are characterized by CIN.

We also determined drug sensitivity after treatment with oxaliplatin, a platinum anticancer agent. Both CT26 and MC 38 cells had inhibited growth in a dose-dependent manner; however, they showed no significant difference in drug sensitivity regardless of mSAT-expression status (IC_50_: control, 0.323 μM vs. mSAT, 0.388 μM in MC38 cells, *p* = 0.600; IC_50_: control, 0.277 μM vs. mSAT, 0.158 μM in CT26, *p* = 0.118, Fig. 4c,d).

### CPT treatment alters levels of apoptosis-related proteins in MC38 cells

**Figure 5:**
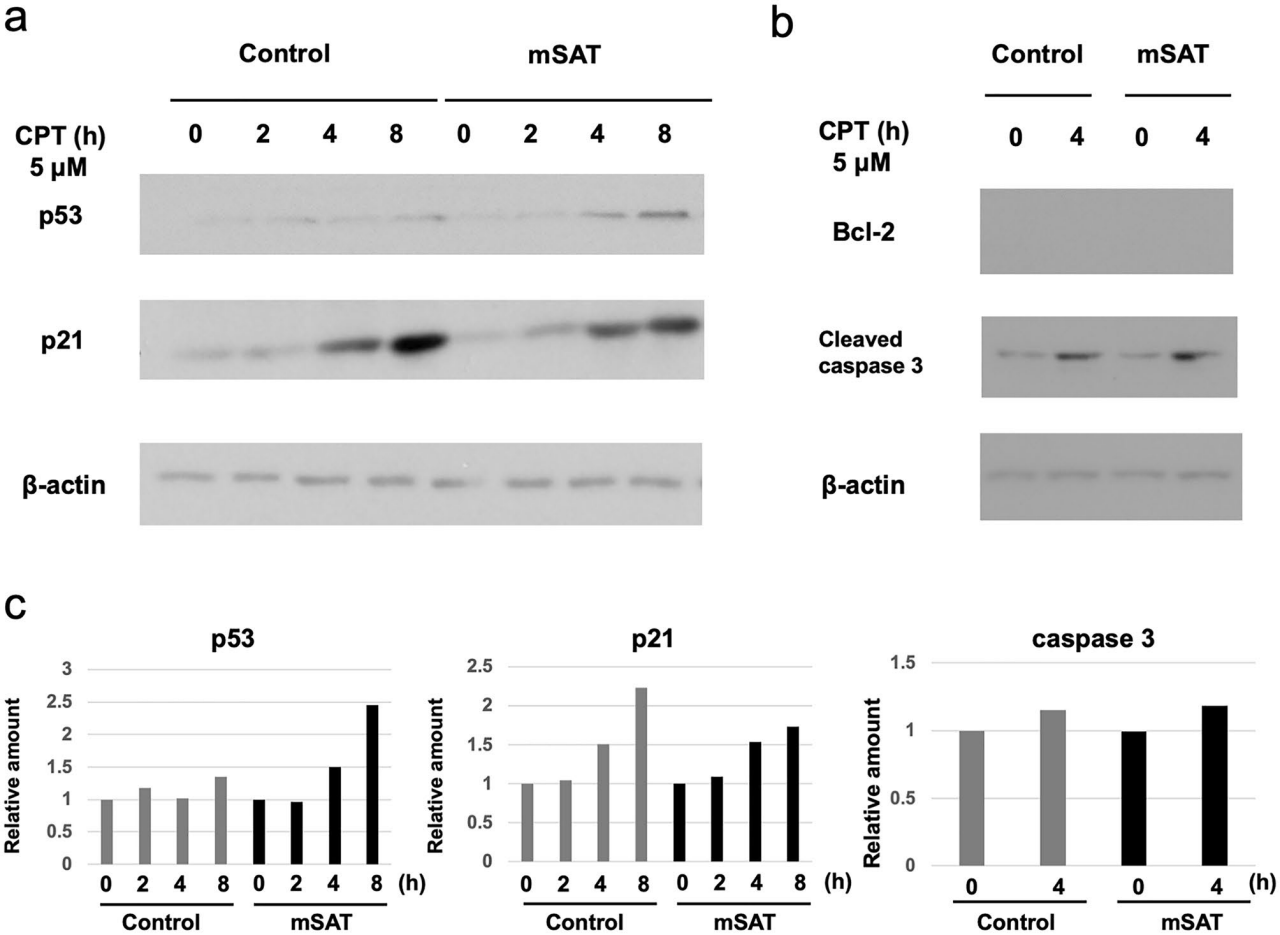
CPT treatment alters the levels of apoptosis-related proteins in MC38 cells. Immunoblot analysis of apoptosis-related proteins following treatment of MC38 cells with 5 μM CPT. (**a**) Treated cells had elevated p53 expression over time, along with the induction of apoptosis, relative to control cells. p21 was expressed over time, with the control cells having higher expression than treated cells. (**b**) Bcl-2 was not expressed. Caspase-3 expression increased over time, along with the induction of apoptosis; however, there was no significant difference between the control cells and those overexpressing mSAT. (**c**) The relative intensities of protein expression determined by Image J are shown. The ratio before CPT treatment was set as 1 in both the control and mSAT cells.

We performed immunoblot analysis to verify the changes in the levels of apoptosis-related proteins and those associated with cellular senescence, such as p53 and p21. Treatment of MC38 cells with and without mSAT overexpression with 5 μM CPT for 2 h, 4 h, and 8 h resulted in significant increases in p53 levels in MC38 cells overexpressing mSAT in a time-dependent manner, whereas these changes were not observed in the control (Fig. 5a). Additionally, we observed significant increases in p21 levels in a time-dependent manner regardless of mSAT-expression status in MC38 cells, suggesting that mSAT overexpression induces cell death rather than cellular senescence in response to CPT treatment (Fig. 5a). Regarding the levels of apoptosis-related proteins bcl-2 and cleaved caspase-3, bcl-2 was not expressed, whereas caspase-3 was expressed in a time-dependent manner; however, no significant difference was observed between the control and mSAT cells (Fig. 5b). Figure 5c shows the relative intensities of protein expression as determined by Image J.

## Discussion

In this study, we demonstrated that mSAT overexpression induces CIN and DNA damage and increases the drug sensitivity of the mouse colon cancer cell line MC38 (MSI) to the topoisomerase I inhibitor CPT. Another mouse colon cancer cell line, CT26 (MSS), did not show a change in CPT sensitivity. These findings may imply that MC38 cells, which are unlikely to harbor CIN, are more susceptible to CIN-induced CPT sensitivity than CT26 cells, which are characterized by CIN. Furthermore, we observed increases in p53 levels but not in those of p21 in CPT-treated MC38 cells, suggesting that mSAT overexpression likely induces cell death rather than cellular senescence in response to CPT.

We found that mitotic errors, including abnormal segregations, micronuclei formation, and anaphase bridging, are enhanced by mSAT overexpression, followed by DNA damage in mouse cell lines, demonstrating that mSAT overexpression induces CIN. Zhu et al.^13^ reported that CRISPR-mediated activation of satellite RNA expression induces CIN in breast cancer cell lines, and Kishikawa et al.^24,25^ showed that mSATs cause chromosomal and genomic instability in a mouse pancreatic cancer cell line. Additionally, we have previously demonstrated that retrovirus-mediated SATs lead to changes in copy number at specific chromosomes in normal human mammary epithelial cells^14^. Overexpression of satellite RNAs is reportedly upregulated in various types of cancers, including those in humans and mice^11^, implying that satellite RNAs that induce CIN are involved in the development of some types of cancer through genomic diversity and heterogeneity^6^.

Multiple studies have reported that drug resistance is induced by CIN^6,15–17^; however, in this study, we found that drug sensitivity is enhanced by treatment with the topoisomerase I inhibitor CPT. This finding is consistent with the increased effect of CPT in cancer cell lines described by Zhang et al.^22^, where CIN reduced the tolerance to genotoxic stress in response to CPT. Additionally, Swanton et al.^17^ demonstrated that sensitivity to carboplatin (a platinum anticancer agent) is increased by CIN but had a reverse effect to taxane (a microtubule-stabilizing inhibitor) in patients with ovarian cancer. CIN is functionally associated with altered intrinsic tumor sensitivity to two distinct drug agents. In this study, drug sensitivity to oxaliplatin (a platinum anticancer agent) was not changed by overexpression of satellite RNAs in mouse colon cell lines, suggesting that CIN-induced drug sensitivity depends on the types of tumor cells. Zhu et al.^13^ demonstrated that satellite RNA-overexpressing cells are more sensitive to DNA-replication stress than DNA double-strand breaks, which is related to our finding that induction of CIN in satellite RNA-overexpressing cells is more sensitive to the DNA-replication stress induced by CPT than the DNA crosslink effects of oxaliplatin. Jamal et al.^18^ showed that extreme CIN predicts improve outcomes in patients with breast cancer, although the mechanisms underlying the induction of drug sensitivity to a specific agent by CIN were not identified. Zaki et al.^20^ reported that widespread DNA damage resulting from lagging chromosomes and chromatin bridges sensitizes tumor cells to additional damage caused by ionizing radiation in combination with 5-FU. Imbalanced genetic stress induced by CIN could contribute to the sensitivity or resistance to each drug.

Disruption of the cell cycle induces cell death, senescence, and altered proliferation. In this study, MC38 cells overexpressing mSAT did not have altered proliferation but had increased cell death, along with increased p53 levels, in response to CPT treatment. Aurora kinase A (AURKA), a cell cycle-regulated kinase involved in spindle formation and chromosome segregation^26^, is associated with CIN in colorectal cancer^27^ and multi-drug resistance in breast cancer^28^. In this study, AURKA levels increased regardless of the presence or absence of mSAT overexpression in response to CPT (Supplementary Fig. 2).

Innate immune signaling can be activated by errors in chromosome segregation and replication stress through the introduction of genomic double-stranded DNA (dsDNA) into the cytosol and engagement of the cyclic GMP-AMP synthase (cGAS)–stimulator of interferon genes (STING) cytosolic dsDNA-sensing antiviral pathway^29–32^. The consequences of CIN not only involve tumor-cell autonomy but also the cross-talk between cancer cells and their immune microenvironment^33^. Although we expected to observe activation of the cGAS–STING pathway by mitotic errors induced by mSAT overexpression, we found that cGAS is expressed before CPT treatment, and that cGAS expression does not increase over time. In contrast, STING was barely expressed in control cells over time but was expressed in mSAT cells before CPT treatment, suggesting that STING might be triggered by mitotic errors induced by mSAT overexpression (Supplementary Fig. 2).

This study has some limitations. First, we used only two colon cancer cell lines as representatives of MSI and MSS cancer; therefore, an in vivo study is required to confirm our in vitro findings. Second, clinical investigations should be conducted in patients with colon cancer treated with CPT by comparing tumor specimens between patients with high expression of human SATs and those with a low expression.

In summary, we demonstrated that the overexpression of a mouse major satellite induces CIN and enhanced drug sensitivity to the topoisomerase I inhibitor CPT in MC38 cells, which are unlikely to harbor CIN and intrinsically resistant to CPT than CT26 cells, which are characterized by CIN. The intrinsic feature of drug resistance to CPT in MC38 cells could be overcome to some extent by satellite RNA overexpression. Our findings offer insight into the different aspects of CIN associated with drug treatment in patients with colon cancer.

## Methods

### Major satellite

TTATGGCGAGGATAACTGAAAAAGGTGGAAAATTTAGAAATGTCCACTCTAGGACGTGGAATATGGCAAGAAAACTGAAAATCATGGAAAATGAGAAACATCCACTTGACGACTTGAAAAATGACAAAATCCCTGAAAAACGTGAAAAATGAGAAATGCACACTGTAGGAGCTGGAATATGGCGAGAAAACTGAAAATCACGGAAAATGAGAAATACACACTTTAGGACGTGAATCTAGCTATGGCGAGGATAACTGAAAAAGGTGGAAAATTTAGAAATGTCCACTCTAGGACGTGGAAAATGGCAAGAAAACTGAAAATCATGGAAAATGAGAAACATCCACTTGACGACTTGAAAAATGACAAAATCACTAAAAAATGTGAAAAATGAGAAATGCACACTGAAGGACCTGGAATATGGCGAGAAAACTGAAATTCACGGAAAATGAGAAATACACACTTTAGGACGTGAAATCGATACCGTCGCATGGGAATAACTTCGTATAGCATACATTATACGAAGTTATGCTGCTTTTTGCTTGTAC.

### Cell culture and generation of satellite RNA-overexpressing cells

We obtained murine colon cancer MC38 cells from Kerafast (Boston, MA, USA) and CT26 (CRL-2639) cells from the American Type Culture Collection (Manassas, VA, USA). HEK293T cells cloned based on their high transfection efficiency were kindly gifted by Professor Hiroshi Itoh, Nara Institute of Science and Technology. MC38 and HEK293T cells were maintained in Dulbecco’s modified Eagle medium supplemented with 10% fetal bovine serum (FBS), 2 mM glutamine, 100 U/mL penicillin, and 100 mL streptomycin. CT26 cells were maintained in Roswell Park Memorial Institute-1640 medium supplemented with 10% FBS, 2 mM glutamine, 100 U/mL penicillin, and 100 mL streptomycin. All cells were cultured at 37 °C in a 5% CO_2_ atmosphere. The plasmid vector p156RRL-EF1a-GFPU3H1MajSat (#41796; Addgene, Watertown, MA, USA) was a gift from Inder Verma and has been previously described^13^.

To investigate whether the overexpression of major satellite RNA reflects drug sensitivity, we constructed retroviral control vectors and vectors expressing mSAT (Supplementary Fig. 1). cDNA of elongation factor 1α (EF1α), the SV40 polyA signal, and major satellites were amplified using polymerase chain reaction (PCR) with PrimeSTAR GXL DNA polymerase (Takara Bio, Shiga, Japan) and inserted into the *EcoR*I and *Sal*I sites of the MSCV-Puro retroviral vector. To prepare the control vector, only EF1α and the SV40 polyA signal were inserted into the said sites of the MCSV puroviral retroviral vector.

To obtain retroviruses, retroviral vectors expressing mSATs and control sequences were transfected into HEK293T cells (1 × 10^6^ cells/60-mm-diameter culture dish), along with helpers, such as pE-Eco and pGP (Takara Bio). After 24 h, the culture medium was replaced with 1.5 mL of fresh culture medium. Secreted retroviruses were harvested every 4 h during the 24- to 60-h post-transfection period, pooled, and stored on ice. Exponentially growing cells (1 × 10^5^ cells/60-mm-diameter culture dish) were infected with 2 mL of virus-containing conditioned medium, along with 1.0 μg/mL polybrene (Merck Millipore, Billerica, MA, USA). After 24 h, the infected cells were cultured in complete medium containing suitable concentrations of puromycin (6 μg/mL for CT26 cells; 4.5 μg/mL for MC38 cells) for 3 days, after which selected cells were used for the subsequent experiments.

### RNA extraction and quantitative reverse transcription (qRT)-PCR

Total RNA was extracted using TRIzol reagent (Life Technologies; Thermo Fisher Scientific, Waltham, MA, USA). To synthesize the single-stranded cDNA, 2 μg of total RNA was added to a 20-μL reaction mixture containing 100 U ReverTra Ace, 1 mM dNTPs, and 5 pmol oligo(dT)_20_ primer (TOYOBO, Osaka, Japan), followed by cDNA synthesis for 60 min at 42 °C. The reaction was terminated by heating at 95 °C for 5 min and diluted with 80 μL TE buffer. Synthesized cDNA (1 µL) was then used for quantitative PCR in a 20-μL volume using the KAPA SYBR Fast qPCR kit (KAPA Biosystems, Wilmington, MA, USA), with the reaction subsequently analyzed using a LightCycler 96 system (Roche Diagnostics, Basel, Switzerland). The PCR primer sequences were used: ubiquitin forward, GGAAGGCATTCCTCCTGAT and reverse, CCCACCTCTGAGACGGAGTA; and major satellite forward, GGCGAGAAAACTGAAAATCACG and reverse, CTTGCCATATTCCACGTCCT.

### Cell proliferation and soft agar colony formation assay

Cells were seeded at 5 × 10^4^ cells/60-mm-diameter dish and counted on days 2 and 4. For the colony formation assay, cells were seeded onto soft agar at 1 × 10^4^ cells/35-mm-diameter dish and grown for 2 to 3 weeks. Visible colonies with a diameter of ≥ 1.0 mm were counted using Image J software (NIH, Bethesda, MD, USA; freely provided by Dr. Wayne Rasband: https://imagej.nih.gov/ij/), with the results shown as a graph.

### Immunocytochemistry

Cells were cultured on cover glasses in well plates. The prepared cover glasses were fixed in 4% paraformaldehyde/phosphate-buffered saline (PBS) at 25 °C for 15 min and washed three times with PBS. The cells were permeabilized and blocked with 0.3% Triton X-100 (Agilent Technologies, Santa Clara, CA, USA) and blocked with 5% FBS at room temperature for 60 min. The cells were then washed three times with PBS and incubated with anti-α-tubulin (1:1000 #T6199 Sigma Aldrich, St Luis, Missouri, USA) and anti-γH2AX (1:500 #2578 Cell signaling Technology, Danvers, MA, USA) at 4 °C overnight. After extensive washing with PBS, the samples were incubated with Alexa-594-conjugated goat anti-mouse IgG secondary antibody (1:500 #A11001 Life technologies,) and Alexa-488-conjugated anti-rabbit IgG secondary antibody (1:500 #A11072 Life technologies) at 25 °C for 60 min. The cover glasses were washed three times with PBS, mounted in in VECRASHELD Mounting medium for fluorescence (Vector Laboratories, Inc Burlingame, CA), and sealed with nail polish. Images were acquired using OLYMPUS FSX 100 fluorescence microscope (OLYMPUS, Tokyo, Japan).

### Drug sensitivity and WST-1 assay

Cells were seeded in microplates (96 wells) at a concentration of 5 × 10^4^ cells/well and 100 μL of culture medium and incubated for 24 h at 37 °C with 5% CO_2_. Irinotecan (CPT; I6932; Funakoshi, Tokyo, Japan) was dissolved in DMSO and added to the medium (to final concentrations of 0, 0.1, 0.3, 1.0, 3.0, and 10.0 μM). Oxaliplatin (156–02,691; Wako, Tokyo, Japan) was added to the medium (to final concentrations of 0, 0.01, 0.1, 1.0, 10.0, and 100.0 μM). The cell growth reagent for the WST-1 assay was added (10 μL/well) and incubated for 30 min at 37 °C with 5% CO_2_ after a 48-h incubation with CPT and 24-h of oxaliplatin. The absorbance of cells between 420 and 480 nm against a blank and background control was measured using a Bio-Rad iMARK Microplate Reader (Bio-Rad Inc. Hercules, CA, USA) .

### Western blot analysis

Cells were lysed with radioimmunoprecipitation assay buffer without sodium dodecyl sulfate (SDS) [10 mM sodium phosphate (pH 7.2), 150 mM NaCl, 3 mM MgCl_2_, 2 mM EDTA, 1% NP-40, 1% sodium deoxycholate, 0.2 U/mL aprotinin, and phosphatase inhibitors] and briefly sonicated on ice. Debris were removed by sedimentation in a microcentrifuge at 16,400 × *g* for 10 min at 4 °C, and the cleared cell lysates were harvested and mixed with Laemmli sample buffer. Proteins (25 µg) from whole-cell lysates were loaded in each lane of an SDS–polyacrylamide gel, separated by electrophoresis, transferred onto polyvinylidene difluoride membranes (Merck Millipore), and visualized by immunoblotting with the indicated antibodies and enhanced chemiluminescence (GE Healthcare, Provo, UT, USA). The relative intensities of protein expression were determined by Image J (http://rsb.info.nih.gov/ij/index.html). The relative ratio was calculated by comparing with the amount before CPT treatment. The ratio before CPT treatments was set as 1 in both the control and mSAT cells.

### Statistical analyses

All statistical analyses were performed using GraphPad Prism (v.9.0; GraphPad Software, San Diego, CA, USA). When necessary, differences in qualitative variables were evaluated using either the χ^2^ test or Fisher’s exact test. Continuous variables were compared using analysis of variance with the Tukey–Kramer test, and the means or medians were compared with the paired samples *t*-test for normally distributed variables. Dose–response curves with the responses normalized to the zero dose as a function of log concentration were generated and statistically compared using the sum-of-squares *F* test. The half-maximal inhibitory concentration (IC_50_) values were obtained using a four-parameter logistic model. Statistica signifcance was set at *p* < 0.05.

## Supplementary Information

Supplementary Information 1.

Supplementary Information 2.

## Data Availability

The datasets that support the findings of this study are available from the corresponding author on reasonable request.
