# Supplementary material for: Overexpression of satellite RNAs in heterochromatin induces chromosomal instability and reflects drug sensitivity in mouse cancer cells
Source: Sci Rep. 2022 Jun 29;12:10999. doi: 10.1038/s41598-022-15071-3 (PMC9243030; doi:10.1038/s41598-022-15071-3)
Supplement: Supplementary file 1 — Supplementary Information 1. [file 41598_2022_15071_MOESM1_ESM.docx]

**Supplementary information for**

**Overexpression of satellite RNAs in heterochromatin induces chromosomal instability and reflects drug sensitivity in mouse cancer cells**

**
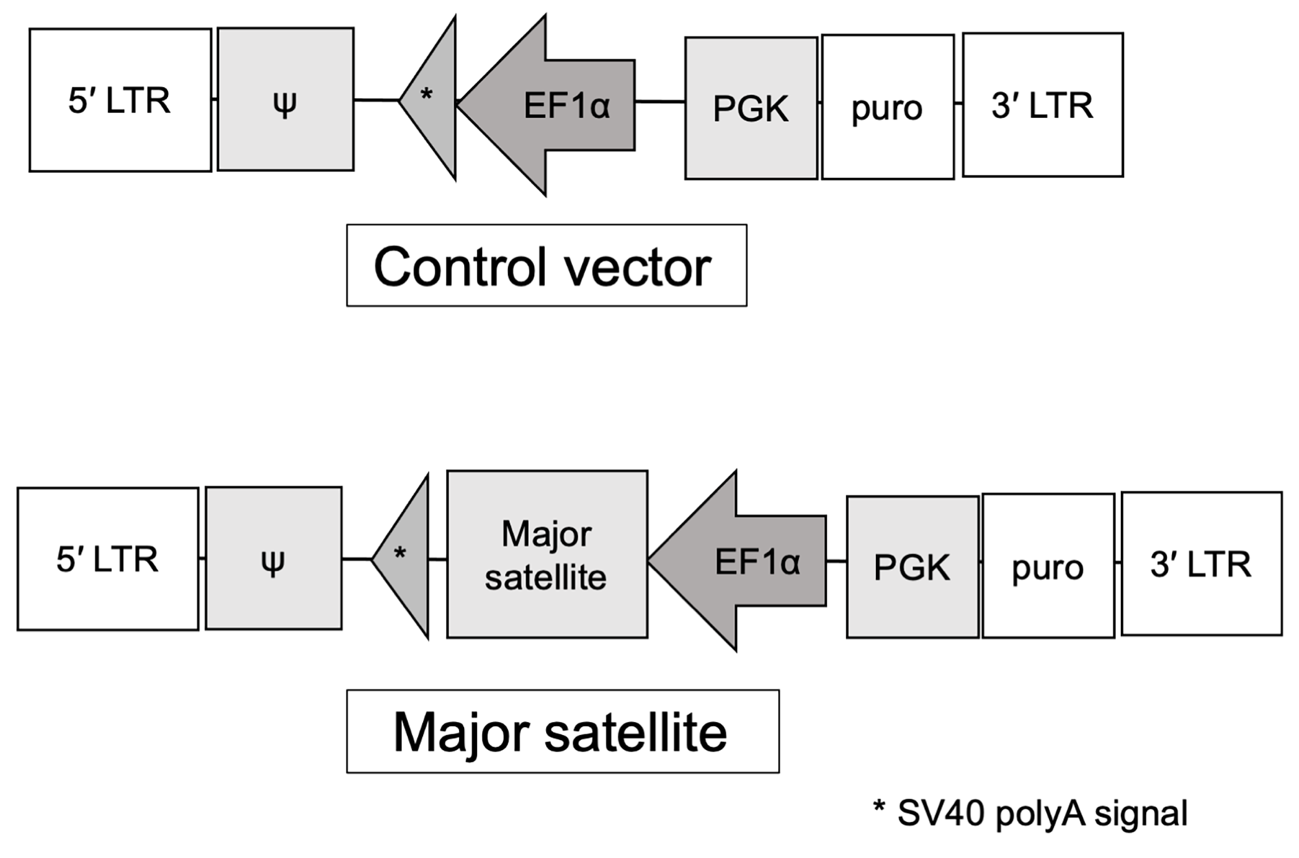
**

**Supplementary Figure 1. Diagrams of the retroviral vector expressing the major satellite and the control vector.**

**
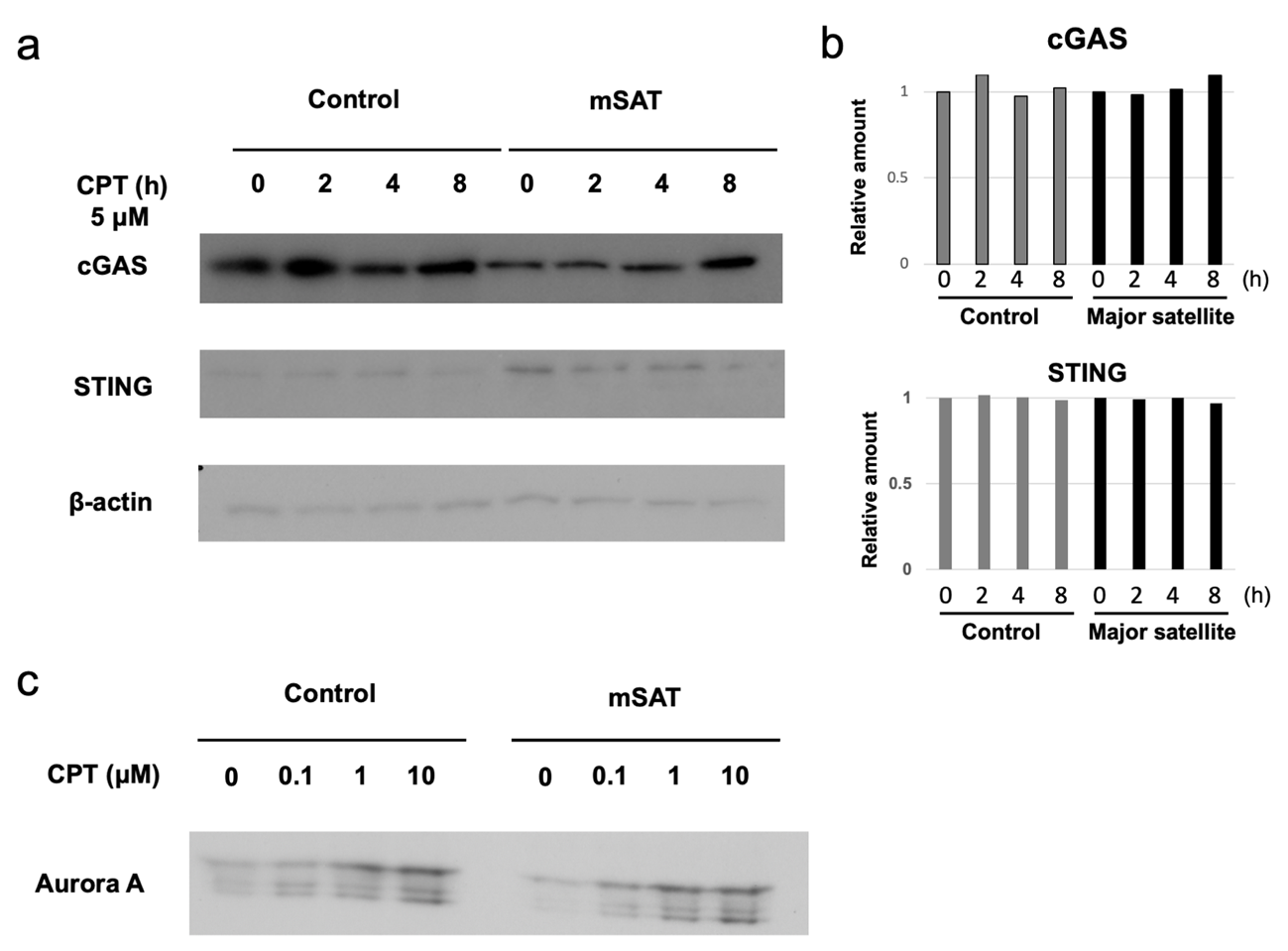
**

**Supplementary Figure 2. Immunoblot analysis of cGAS, STING and Aurora A, following treatment of MC38 cells with CPT.**

(a) Immunoblot analysis of cGAS and STING. cGAS was expressed before CPT treatment, and cGAS expression did not increase over time. In contrast, STING was barely expressed in control cells over time but was expressed in mSAT cells before CPT treatment, suggesting that STING might be triggered by mitotic errors induced by mSAT overexpression. (b) The relative intensities of protein expression determined by Image J are shown. The ratio before CPT treatments was set as 1 in both the control and mSAT cells. (c) Immunoblot analysis of Aurora A. Treated cells had elevated Aurora A expression as the concentration of CPT increases; however, no significant difference was observed between the control and mSAT cells.
