# Supplementary material for: Overexpression of satellite RNAs in heterochromatin induces chromosomal instability and reflects drug sensitivity in mouse cancer cells
Source: Sci Rep. 2022 Jun 29;12:10999. doi: 10.1038/s41598-022-15071-3 (PMC9243030; doi:10.1038/s41598-022-15071-3)
Supplement: Supplementary file 2 — Supplementary Information 2. [file 41598_2022_15071_MOESM2_ESM.docx]

**Supplementary information for**

**Overexpression of satellite RNAs in heterochromatin induces chromosomal instability and reflects drug sensitivity in mouse cancer cells**

**Uncropped data of western blots for Figure 5 and Supplementary Figure 2**


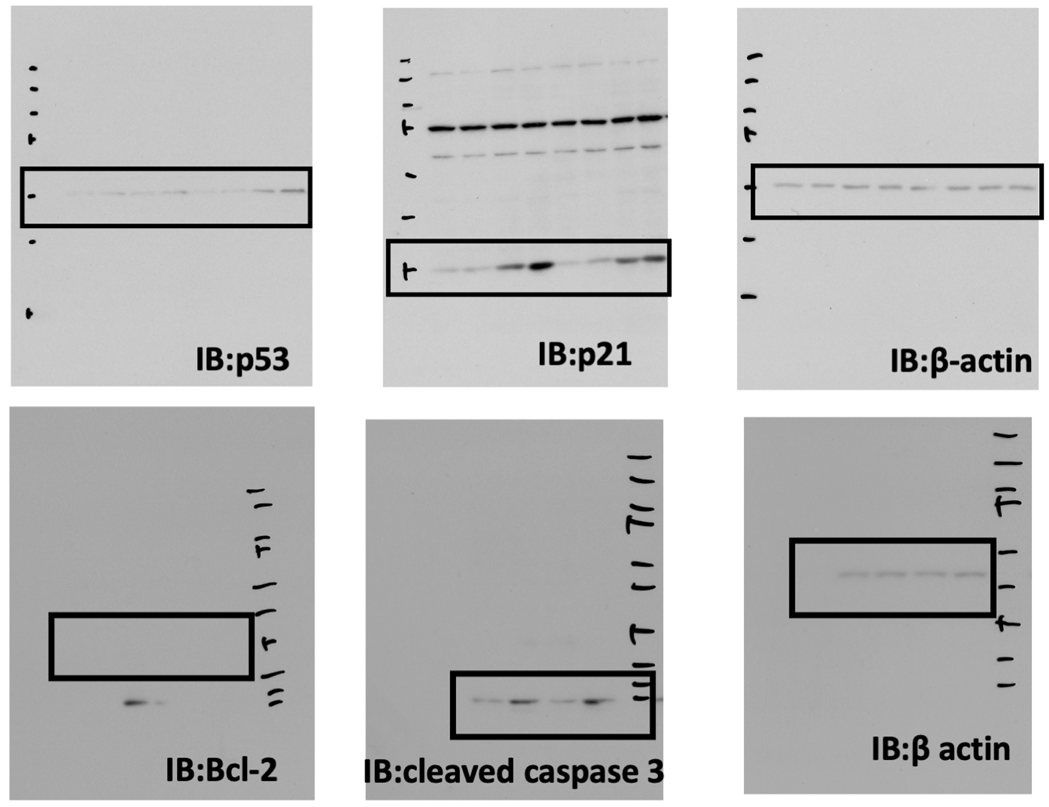


**Figure 5**


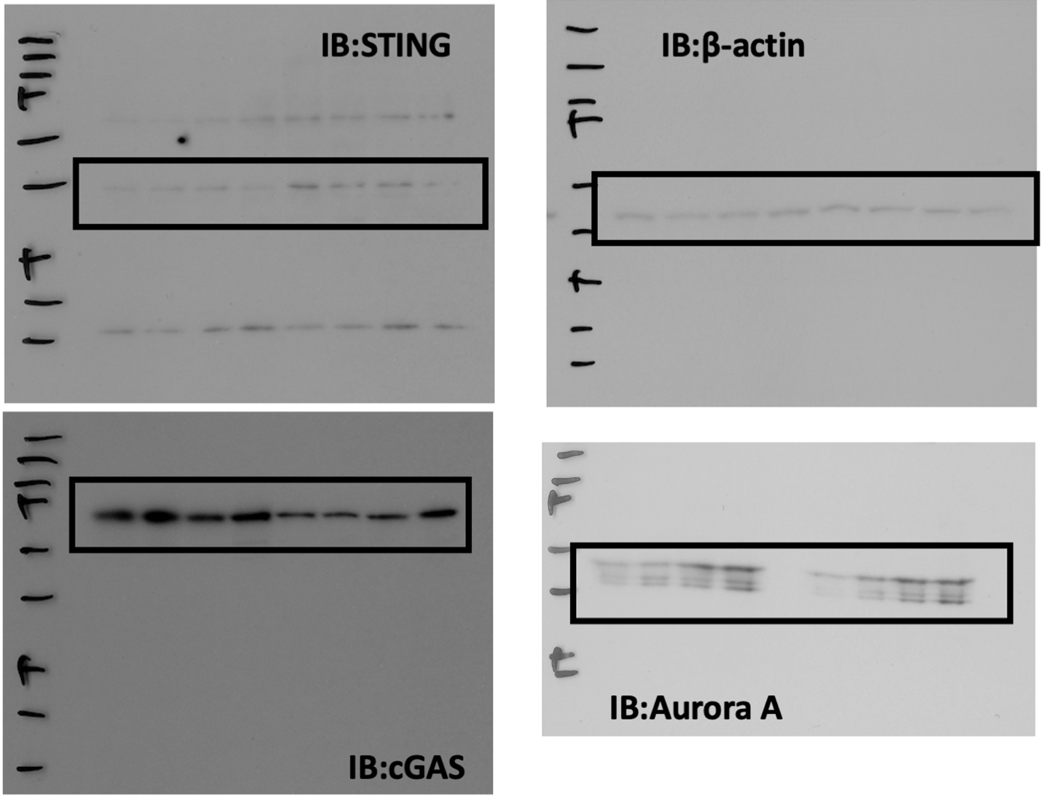


**Supplementary Figure 2**
